# Effect of rTMS on Parkinson’s cognitive function: a systematic review and meta-analysis

**DOI:** 10.1186/s12883-020-01953-4

**Published:** 2020-10-19

**Authors:** Yi Jiang, Zhiwei Guo, Morgan A. McClure, Lin He, Qiwen Mu

**Affiliations:** 1 Department of Radiology and Institute of Rehabilitation and Imaging of Brain Function, The Second Clinical Medical College of North Sichuan Medical College Nanchong Central Hospital, NO. 97 South Renmin Road, Shunqing District, Nanchong, 637000 Sichuan China; 2 Department of Radiology, Peking University Third Hospital, Beijing, China

**Keywords:** Transcranial magnetic stimulation, Parkinson’s disease, Cognitive deficit, Executive function

## Abstract

**Background:**

To evaluate the effects and optimal parameters of repetitive transcranial magnetic stimulation (rTMS) on cognition function of patients with Parkinson’s disease (PD) and to estimate which cognitive function may obtain more benefits from rTMS.

**Method:**

The articles dealing with rTMS on cognitive function of PD patients were retrieved from the databases until April 2019. Outcomes of global cognitive function and different cognitive domains were extracted. The standardized mean differences (SMDs) with 95% confidence interval (CI) of cognitive outcome for different parameters, scales, and cognitive functions were estimated.

**Results:**

Fourteen studies involving 173 subjects were included in this meta-analysis. A significant effect size was observed with the mini-mental state examination (MMSE) for the global cognitive outcome based on the evidence of four published articles. Further subtests for different cognitive domains demonstrated prominent effect for the executive function. The significant effect sizes for executive function were found with multiple sessions of high-frequency rTMS over frontal cortex; especially over dorsolateral prefrontal cortex (DLPFC). All of the other cognitive domains, which included memory, attention, and language ability, did not obtain significant effects.

**Conclusions:**

Multiple sessions of high-frequency rTMS over the DLPFC may have positive effect on executive function in PD patients. Further well designed studies with large sample sizes are needed to verify our results and ascertain the long-term effects of rTMS.

## Background

Parkinson’s disease (PD) is the second largest progressive neurodegenerative disease except Alzheimer’s disease (AD). In addition to motor symptoms such as bradykinesia, rigidity, postural instability, and gait disturbances, PD patients are usually accompanied by a series of non-motor symptoms (NMS) such as depression, cognitive dysfunction, and autonomic dysfunction [1]. From previous epidemiological data, the NMS of PD are under-reported, but they serve as a key determinant of quality of life and occur across all stages of PD [2]. Cognitive dysfunction, the most common and probably most devastating of NMS, results in a spectrum of deficits ranging from MCI to severe dementia [3] and can present early in the disease course. A recent review [4] has shown that 21.0% of their PD sample met criteria for PD-MCI, and 17.0% had dementia. Nearly 80.0% of MCI patients eventually develop dementia in the later stage of the disease, and dementia is an important and independent predictor of mortality in patients with PD [5, 6]. The forms of cognitive deficit in PD patients vary including executive dysfunction, visual spatial disorder, memory decline, and language dysfunction. Among them, executive function impairment has been the most prominent as a clinical practical multi-center collaborative study [7] found executive dysfunction accounted for 10.1% in PD patients with cognitive deficit. However, due to the lack of understanding and neglect of the physiological processes mediating cognitive changes by clinicians, the underlying pathogenesis and mechanism of cognitive deficit in PD patients are still unclear, but they are closely related to the complex neuropathological abnormalities of PD [8]. For instance, in the brain of patients with PD, neurotransmitters are changed, dopminergic neurons in the substantia nigra are lost, striatum dopamine is depleted, and the cortical-subcortical dopamine loop between basal ganglia and frontal lobe is significantly damaged [9, 10]. Beyond that, the atrophy of the hippocampus and frontal cortex as well as the precipitation of abnormal proteins in PD patients may contribute to cognitive deficit. At present, there is no cure for PD. Traditional treatments such as physical exercises, pharmacotherapy, and cognitive therapy have some benefits for patients’ life. However, some of these therapies may cause a series of side effects such as nausea, vomiting, and aggravating symptoms of exercise [11]. Therefore, identifying safe therapies to alleviate symptoms remains a priority.

In recent years, based on the reporting guidelines established by a group of European experts on the therapeutic use of repetitive transcranial magnetic stimulation (rTMS) and on evidence published until 2018, rTMS is recommended as a potential therapeutic tool for various neurological and psychiatric disorders [12]. rTMS is a painless, non-invasive, well-tolerated technique of brain stimulation based on the theory of electromagnetic induction [13]. It can induce currents in the local areas of cerebral cortex through rapidly changing magnetic fields to depolarize nerve cells of central nervous system and produce activity of the synaptic terminals, which may lead to a series of brain metabolic changes and other physiological functional responses [14]. Long term of rTMS has cumulative effect on the brain, some of which might depend on long-term potentiation (LTP)/long term depression (LTD)-like changes in synaptic connections between cortical neurons [15]. This has been reported by some studies in various neurological, psychiatric, and neuropsychiatric diseases and even in healthy controls [16–19]. The nature of the after-effects of rTMS depends on the stimulation site, pulse number, stimulation intensity, frequency, and the number of treatment sessions [15]. For example, stimulation at frequencies higher than 1.0 Hz tends to increase rather than decrease cortical excitability [20]. However, due to the lack of understanding on the mechanism of sustained repair of cortical excitability caused by stimulation, and the variability of the within-subject and between-subject induced by rTMS, there is no consensus on rTMS parameters and overall efficacy.

In 2014, Anderkova et al. [21] preliminarily reviewed the application of rTMS on cognitive impairment in PD patients, AD patients, and MCI patients from a clinical perspective. This study reported the after-effects of rTMS and its variability due to distinct stimulation magnitude, protocols, stimulated areas, control procedures, and neuropsychological methods for assessment of after-effects. In 2017, another study [22] reviewed the potential therapeutic effects of non-invasive stimulation including rTMS and transcranial direct current stimulation (tDCS) on depression and NMS in PD patients and showed that rTMS had some positive effects on depressive symptoms and cognitive impairment in PD patients. Besides, Dinkelbach’s [23] suggested the importance of rTMS stimulation sites, in which the dorsolateral prefrontal lobe (DLPFC) was considered as a crossroads of depression and cognitive function. Randver’s study [24] pooled the available rTMS studies on NMS of PD with the stimulation site of DLPFC, i.e., mood disturbance and cognitive impairment, which showed that high-frequency rTMS was beneficial for PD-related depression, but the availability on reducing PD-related cognitive impairment has remained uncertain. In 2017, Lawrence et al. [25] performed a detailed analysis of rTMS on different cognitive domains of PD patients with three included studies, which reported a negative outcomees. Another meta-analysis that conducted by Goodwill et al. [26] with five articles also didn’t found any significant effect for stimulation parameters on cognitive function. Although quantitative analysis was performed in these two studies, there is no further subgroup analysis for different rTMS parameters. Subsequently, in 2018, both Cohen et al. [27] and Buard et al. [28] published related studies. Therefore, the efficacy of rTMS on cognitive function of PD remains controversial and the optimal parameters are still unclear. To resolve these issues, the purpose of this study was to provide an objective and comprehensive analysis that whether rTMS treatment was effective on cognitive function of PD patients, which cognitive domain obtained more from rTMS stimulation, and which rTMS parameters are the most appropriate.

## Method

The meta-analysis was conducted based on the Preferred Reporting Items for Systematic Reviews and Meta-analyses (PRISMA) statement.

### Search strategy

We performed the meta-analysis with the data from the PubMed, Cochrane Library, Embase, Sciencedirect, and Web of science published before April 2019. In order to collect the literature comprehensively, a wide range of terms were used. These were “rTMS” or “TMS” or “magnetic stimulation” or ‘repetitive transcranial magnetic stimulation’, “cognitive” or “cognition” or “MCI” or “mild-cognitive impairment” or “neurodegenerative”, and “Parkinson” or “PD”.

### Inclusion and exclusion criteria

The included studies strictly meet the following inclusion criteria: application of rTMS, involvement of PD patients, measurement of the cognitive function (i.e. memory, execution, attention, language function, and global cognitive function), and published in English. Exclusion criteria: multiple combined interventions, insufficient data (no original outcome data or their mean and standard deviations (SD) values which can be used in the meta-analysis were provided), no standardized cognitive outcome, study protocol (only design scheme, no specific data and results), and case-report studies. In order to include all relevant articles more broadly, the design of the study was not limited. Studies with randomized controlled trials (RCT), crossover trials, and self-controlled design were all included in the search.

### Study quality

The checklist from Moher et al. [29] was modified to assess the quality of the included trials. Briefly, the following criteria was used to evaluate the quality of each trial: 1) whether the experimental design was randomized; 2) whether the blind method was adopted and the type of the blind method was recorded in detail; 3) During the experiment, whether the subjects drop out, if so, whether the number of drop out is recorded in detail; 4) whether the detailed basic information of the subjects was included; 5) whether the experiment was a comparison between the control group and the experimental group; and 6) whether any adverse reactions were reported, and if any, the number of adverse reactions, the type, and severity of adverse reactions were described in the article.

### Data extraction

Two experienced reviewers independently evaluated the studies based on the inclusion/exclusion criteria. Any disagreement was resolved through discussion and consultation with a third reviewer. The detailed basic information was extracted which included the first author, release year, intervention method, the course of the disease, number of subjects, and rTMS parameters (Table 1). In order to reduce the heterogeneity produced by different experimental designs including RCT, crossover trials, and self-controlled trials, we only extracted the mean and SD of the cognitive scale data before and after rTMS treatment. If the standard error of the mean (SEM) was provided, it was converted to SD by using the formula of SD = SEM× $\sqrt{n}$. Due to multiple domains of the cognitive function, a single scale to detect rTMS treatment for cognition may not be meaningful. Duchek [41] proposed that cognitive dysfunction may include the impairment of the following cognitive aspects: language understanding, language generation, pattern recognition, task organization, reasoning, attention, and memory. Base on the main aspects involved in the intervention, Cicerone et al. [42] classified cognitive rehabilitation into seven categories: attention, visual perception, visual constructive ability, verbal communication, memory, question resolution, executive function, multiple model cognitive impairment, and comprehensive cognitive rehabilitation. Except for these seven cognitive categories, another recent study of this author [43] believed that integration of individualized cognitive also could be considered as a cognitive category. Therefore, based on the existed classification and cognitive scales mentioned in the included studies, four categories involving executive function, memory, attention, as well as language function were analyzed in this meta-analysis. The response time and accuracy of the different cognitive tasks were recorded to combine the results to reflect the therapeutic effect of rTMS. In addition, to avoid the heterogeneity caused by the diversity of the scale, we tried to unify the scale in the same cognitive field when multiple cognitive tests were applied in the study.

**Table 1:** Characteristics of Included Studies

Author/Year	Subject	Age	Duration of disease	Stimulation Parameters			
				Position	Session	Frequency	Intensity
Boggio 2005 [30]	13	65.2 ± 1.65	6.7 ± 0.92	Left DLPFC	10	15 Hz	110% RMT
Cardoso 2007 [31]	11	67 ± 8.3	11 ± 7.6	Left DLPFC	12	5 Hz	120% RMT
Epstein 2007 [32]	14	62.0	/	Left DLPFC	10	10 Hz	110% RMT
Benninger 2009 [33]	9	62.6 ± 9.6	/	Left M1	1	50 Hz	60% RMT
Toshiaki 2009 [34]	6	66.8 ± 3.4	7.17 ± 3.1	Frontal region	12	0.2 Hz	120% RMT
Sedlkov 2009 [35]	10	63.7 ± 6.7	7.8 ± 6.5	Left PMd	1	10 Hz	100% RMT
				DLPFC	1	10 Hz	100% RMT
				OCO	1	10 Hz	100% RMT
Pal 2010 [36]	12	68.5 ± 7.9	6	Left DLPFC	10	5 Hz	90% RMT
Kimura 2011 [37]	12	69.2 ± 5.8	8.5 ± 4.4	SMA	4	0.2 Hz	/
Srovnalova 2011 [18]	10	66 ± 6	5.4 ± 2.45	Left and right inferior frontal gyri	1	25 Hz	80% RMT
Srovnalova 2012 [38]	10	66.0 ± 6	5.4 ± 2.45	Left DLPFC	4	25 Hz	80% RMT
			/	Right DLPFC	4	25 Hz	80% RMT
Chang 2017 [39]	16	63.8 ± 8.3	9.1 ± 5.3	M1-LL	5	10 Hz	90% RMT
Dagan 2017 [40]	7	74.6 ± 7.1	10.3 ± 3.8	mPFC	16	10 Hz	100% RMT
Buard 2018 [28]	22	/	/	Bilateral DLPFC	10	20 Hz	/
Cohen 2018 [27]	21	64.4 ± 6.8	4.7 ± 3.4	M1 + PFC	24	1 Hz + 10 Hz	110% + 100% RMT

*Exp* Experimental Group, *Ctr* Control Group, *SMA* Supplementary Motor Area, *DLPFC* Dorsal Lateral Prefrontal Cortex, *PFC* Prefrontal Cortex, *M1* Primary Motor Cortex, *mPFC* Medial Prefrontal Cortex, *PMd* Dorsal Premotor Cortex, *OCO* Occipital Cortex, *M1-LL* Primary motor cortex of the lower leg; /: no report

### Statistical analysis

The meta-analysis was conducted by using RevMan 5.3 software provided by Cochrane collaboration (London, UK). The standardized mean difference (SMD) and its 95% confidence interval were selected to display the combined results. The heterogeneity was tested by using the Cochran’s Q statistics and I^2^ test. If the I^2^ value was greater than 50%, the random effect model was used for the analysis. Otherwise a fixed model was used. Moreover, the data extraction method and the raw data included in the study was examined by analyzing the clinical intervention measures and experimental design, and by using sensitivity analysis as well as other methods to find the cause of heterogeneity. Inverted funnel diagram was used to assess possible publication bias. In addition, some subgroup analyses were conducted to assess the influence of moderator variables of rTMS on cognitive function. Comparison of outcome variables used a *P* < 0.05 value for statistical significance.

## Results

### Characteristics of the included research literature

**Fig. 1:**
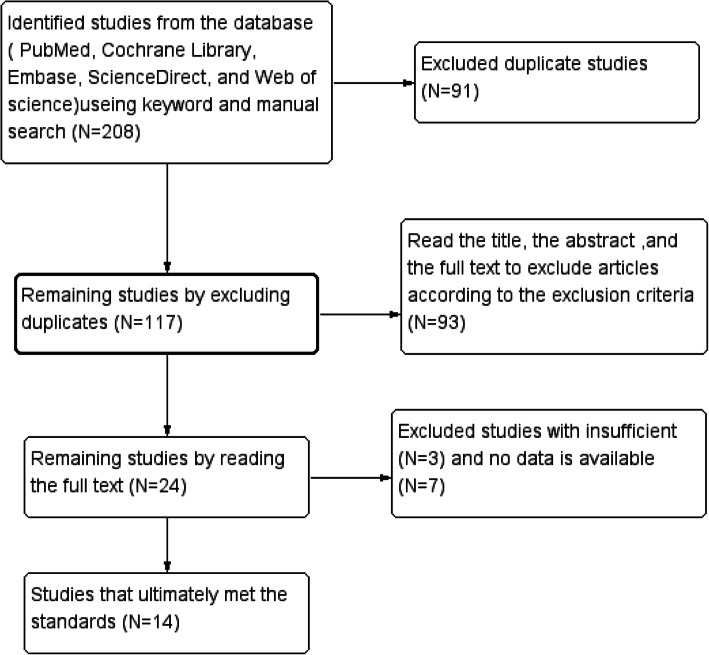
Study screening flow chart

The computer retrieved 208 articles. After reading the title and abstract and excluding duplicate documents, 14 documents were finally left that met the inclusion criteria [18, 27, 28, 30–40]. The detailed article screening process was shown in Fig. 1. A total of six parallel design experiments, five cross-design experiments, and three self-control design experiments were included in this analysis. Seven articles were evaluated for overall cognitive function and 13 articles for different cognitive domains (e.g., executive function, memory, attention, and language function). All articles contain data on immediate efficacy after treatment while only three articles had follow-up data [30, 34, 36].

### Characteristics of the patients with PD

A total of 249 PD patients were enrolled in the 14 studies, but, in this meta-analysis, only the data of patients from the real rTMS group was extracted. Therefore, only 173 PD patients were included in our analysis. A total of 70 subjects suffered from depression including major or minor depression (for the number of patients with different degrees of depression, some authors did not provide the detail description). Three articles included a total of 46 patients with idiopathic PD. Most studies had no detailed information on the patient’s motor syndrome such as tremor, Bradykinesia, or posture gait abnormalities. Six patients suffered from dementia. The average age of the subjects included in 12 articles is over 60 years old, and the average age of the subjects included in one article is over 70 years old. One article did not provide the average age of the subjects. The mean disease duration of almost all patients was more than 5 years, and even the mean disease duration of individual patients was more than 10 years. As shown in Table 1. Most patients had stable medication for a period of time before and during treatment, but the studies lacked any detailed information on drugs.

### Document quality evaluation result

Randomized allocation was used in 11 studies, but detailed distribution method was not mentioned in the other three studies. Seven articles were double-blinded, four studies were single-blinded, and three studies did not report blind-related information which was defined as an unclear blinded method. Four studies documented the number of dropouts, and the remaining ten studies did not report any dropouts if they occurred or not. Almost all articles contained complete patient information such as age, duration of illness, and education level. Adverse reactions were reported in eight studies, two of which definitely reported the number, mild headache was the main side effect, and the rest did not report the number. No adverse reactions were reported in the remaining six studies. No serious side effects were reported in any of the included articles (Table 2).

**Table 2:** Quality assessment of included literatures

Study	Randomization	Blinding	Dropout	Description of basic features	Control study	Adverse events
Boggio 2005 [30]	yes	double	0	yes	yes	0
Cardoso 2008 [31]	yes	double	0	yes	yes	yes^a^
Epstein 2007 [32]	unclear	unclear	2	yes	no	0
Benninger 2009 [33]	unclear	unclear	1	yes	no	0
Toshiaki 2009 [34]	yes	unclear	0	yes	no	0
Sedlkov 2009 [35]	yes	single	0	yes	yes	0
Pal 2010 [36]	yes	double	0	yes	yes	yes^a^
Kimura 2011 [37]	unclear	double	0	yes	yes	0
Srovnalova 2011 [18]	yes	single	0	yes	yes	2
Srovnalova 2012 [38]	yes	single	0	yes	yes	2
Chang 2017 [39]	yes	double	0	yes	yes	yes^a^
Dagan 2017 [40]	yes	single	2	yes	yes	yes^a^
Buard 2018 [28]	yes	double	2	yes	yes	yes^a^
Cohen 2018 [27]	yes	double	0	yes	yes	yes^a^

yes^a^ = Unclear the exact number

### Efficacy evaluation of rTMS on overall cognitive function

**Fig. 2:**
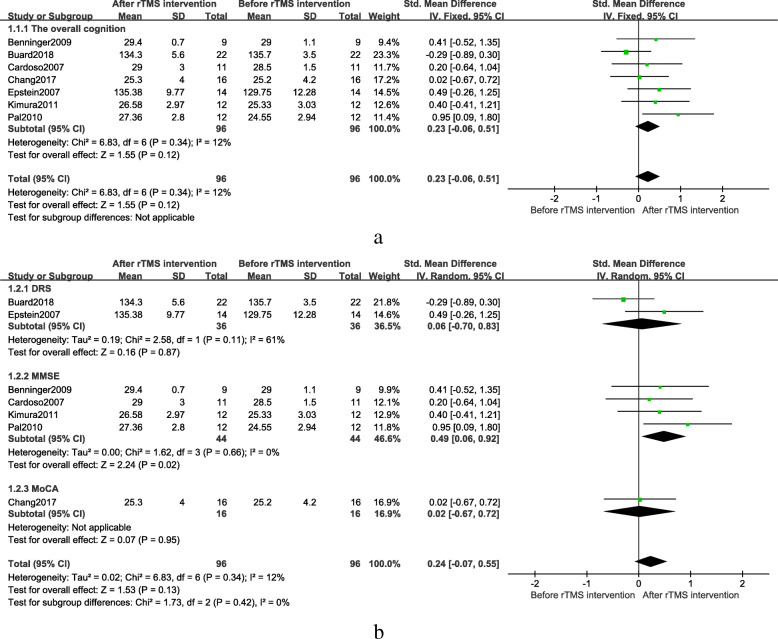
**a** Overall cognitive efficacy after rTMS treatment, **b** Over all cognitive different scales subgroup efficacy after rTMS treatment

Seven studies, which included 96 subjects, evaluated the efficacy of rTMS on the overall cognitive function. Fixed effect mode combined results showed that rTMS treatment improved cognitive function, but did not achieve significant results (SMD = 0.23, 95% CI, − 0.06 to 0.51, *P* = 0.12) (Fig. 2a). Figure 2a indicated that the inconsistency of the overall cognitive scale may lead to deviations on results. Therefore, the subgroup analysis with different overall cognitive scales including the Mattis Dementia Rating Scale (DRS), the Mini-Mental State Examination (MMSE), and the Montreal Cognitive Assessment (MoCA) was conducted.

### Different scale subgroup results

The scale subgroup analysis with random effect model showed that the MMSE group had significant results without heterogeneity (SMD = 0.49, 95% CI, 0.06 to 0.92, *P* = 0.02). Figure 2b showed the results of the two sets of scales which were distinctly different, indicating the choice of the scale may result in a certain deviation.

### rTMS treatment for different cognitive domains

**Fig. 3:**
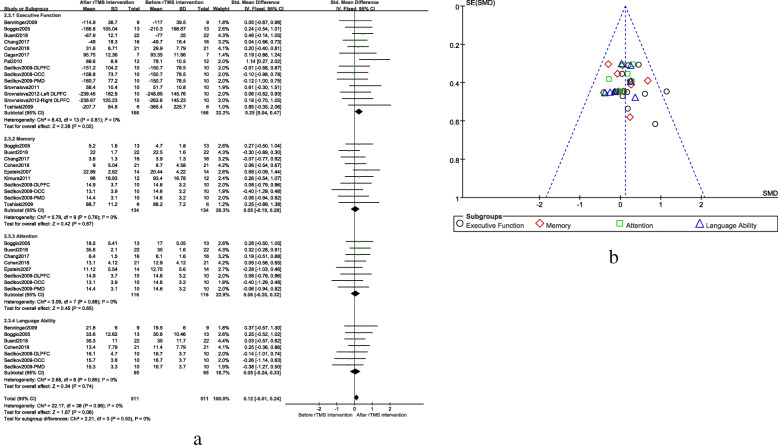
**a** Therapeutic effects of different cognitive domains after rTMS treatment, **b** Publication biased funnel plots in different cognitive domains

The data from four cognitive domains including executive function, memory, attention function, and language function were analyzed. Among them, 11 studies related to executive function included 166 patients; eight studies related to memory included 134 patients; six studies related to attention included 116 patients; and five studies related to language function included 95 patients. The fixed effect model combined results showed a significant improvement on executive function after rTMS (SMD = 0.25, 95% CI, 0.04 to 0.47, *P* = 0.02), but no significant results were found in other cognitive domains. The result of the funnel plot (Fig. 3a) showed that the left and right were basically symmetrical indicating there was no or slight publication bias. The result was shown in Fig. 3b.

### Subgroup results on the executive function

**Fig. 4:**
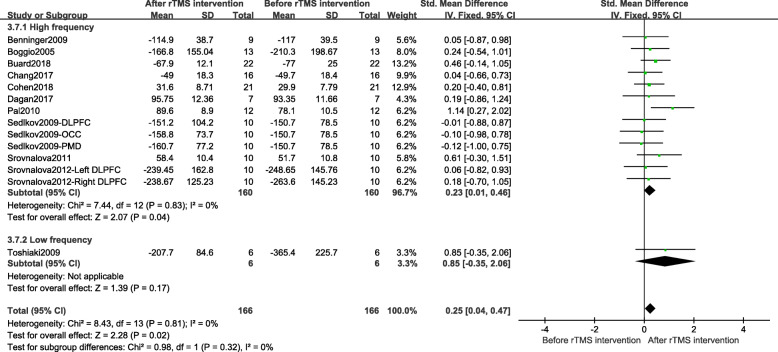
Stimulation frequency subgroup results after rTMS on executive function

**Fig. 5:**
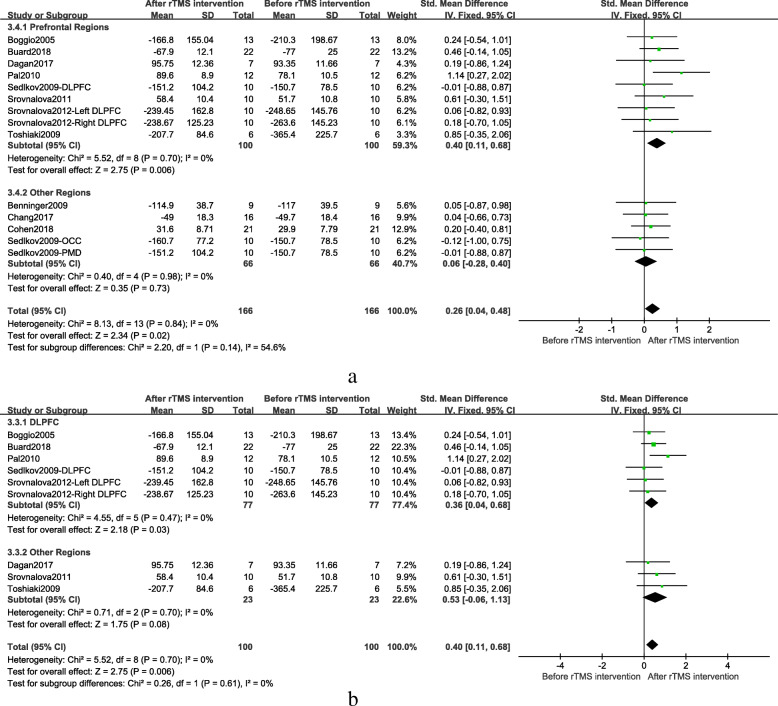
**a** Stimulation site subgroup (the frontal region vs other regions) results after rTMS on executive function, **b** Stimulation site subgroup (the DLPFC group vs other frontal regions group) results after rTMS on executive function

**Fig. 6:**
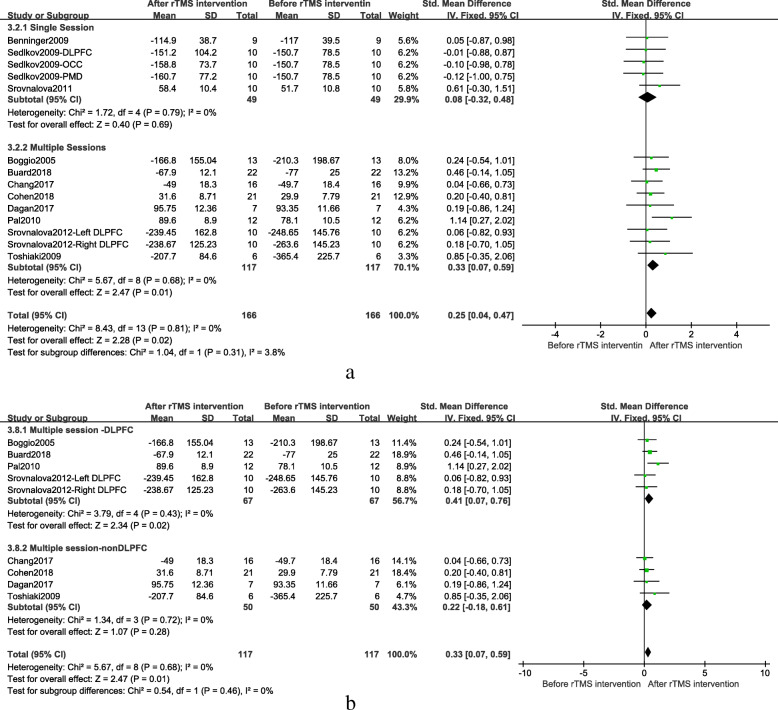
**a** Stimulation session subgroup results after rTMS on executive function, **b** Stimulation site subgroup (the DLPFC group vs other regions group) after multiple sessions rTMS on executive function

Since there was almost no heterogeneity among groups in different cognitive domains, only a subgroup analysis of executive function was performed, mainly based on different treatment parameters, including frequency, the treatment site, and the session of treatments. Generally speaking, the frequency was divided into high-frequency (> 1.0 Hz) and low-frequency (≤ 1.0 Hz). Sensitivity analysis found that, after removing low-frequency stimulus document, the combined results of high-frequency stimulation still had a significant effect (SMD = 0.23; 95% CI, 0.01 to 0.46; *p* = 0.04), as shown in Fig. 4. Based on the intimate connection between the frontal area and cognitive function, the treatment site was divided into two groups: the frontal region and other regions. The fixed effect model combined results showed the frontal region group had significant results after rTMS treatment when compared to other regions group (SMD = 0.40; 95% CI, 0.11 to 0.68; *P* = 0.006), as shown in Fig. 5a. The DLPFC is closely related to executive function [44]. In order to get more accurate brain localization, the subgroup analysis of DLPFC and other frontal region was performed. The fixed effect model combined results showed compared to other frontal regions group, the DLPFC group had more significant results (SMD = 0.36; 95% CI, 0.04 to 0.68; *P* = 0.03). Details were shown in Fig. 5b. The session of treatments can be divided into two groups: single session treatment and multiple session treatments. The fixed effect model combined results showed multiple session treatments were significantly effective after rTMS treatment (SMD = 0.33; 95% CI, 0.07 to 0.59; *P* = 0.01). Details were shown in Fig. 6a. In addition, the result of multiple sessions was still significant when only the studies on DLPFC were used (SMD = 0.41; 95% CI, 0.07 to 0.76; *P* = 0.02). Details were shown in Fig. 6b.

## Discussion

In this study, we quantitatively tested the efficacy of rTMS on cognitive functions of PD patients. On the whole, the results of the published works showed positive effect of rTMS, mainly in specific tasks MMSE, which had a significant performance, suggesting the effect of rTMS on patients was associated to task-specific cognitive improvement. Moreover, the stratified results showed the high frequency rTMS stimulation over the DLPFC for multiple sessions had a significant performance on executive function of PD patients, but in other cognitive domains, no positive performance was found.

In 2017, the meta-analysis of Lawrence et al. [18] showed that cognitive function did not appear to be improved after rTMS, but, this article only enrolled three rTMS studies which investigated different cognitive domains involving global cognition, executive function, and attention respectively. In our analysis, the subgroup analysis was also performed in which one study was included for global cognition, two studies for executive function, and one study for attention. In order to get preliminary results of rTMS trials in cognition in PD, more detailed exploration of this therapeutic technique should be required. Goodwill et al. showed a negative result, based on the evidence of five published articles. There were obvious shortcomings in the results. For example, the refinement of the cognitive domain was unreasonable (the integration of executive function and psychomotor speed is analyzed as global cognition). The results of these measures should be taken with caution. In our study, 14 studies showed that rTMS treatment played a positive role in the improvement of cognitive function, which was similar to previous reviews of Anderkova et al. [22] and Dinkelbach et al. [23] in 2017. In fact, the efficacy of rTMS on cognition was reported in other neuropsychiatric disorders meta-analysis, such as, in both dementia [45] and schizophrenia [16] studies. Subgroup results of overall cognitive function showed cognitive improvement in the MMSE group was significantly better than that in the DRS group and the MoCA group. This revealed that the specificity of the task results in deviation in outcomes. Because MoCA scale was used in one study, in order to avoid the result deviation caused by small sample size, only MMSE scale and DRS scale were discussed. The MMSE scale contains 10 aspects of cognition: orientation, instant memory, attention and computational power, delayed recall, object naming, language retelling, and speech comprehension. From the design of the content of the MMSE scale, it contains a large proportion of the evaluation of the orientation force (10/30 points). The DRS scale contains five aspects of cognition: attention, start and hold, concept formation, structure, and memory. From the content design of the scale, the concept formation accounts for a large proportion (39/144 points). Each global cognitive scale has slightly laterality for distinct cognitive field. The complaints of typical PD-MCI patients generally include slower work, decreased concentration, and vocabulary search barrier. The most prominent of the damaged cognitive domains found in PD-MCI patients is the ability of executive functions, attention, and orientation, etc. [4] In addition, DRS is significantly affected by age and education level, while MMSE is not directly related to these factors. But, in general, DRS is more sensitive and specific for cognitive impairment assessment than MMSE. Even Monsch et al. [46] found the DRS is a clinically valid psychometric test for the detection of dementia patients in which the Memory and Initiation/Perseveration subscales are its best discriminative indexes. Besides, the low sensitivity of MMSE scale and the impact of low sample size (only four articles for MMSE) also should not be neglected. Therefore, this result still needs to be treated with caution. The Movement Disorder Society Task Force recommended a series of neurocognitive scales to define PD-MCI [47], but the number of scales involved is too large to conducive to rapid screening. Ideal PD cognitive function evaluation tools should conform to the following criteria. 1. Covering subcortical and cortical dementia detection items. 2. High sensitivity and specificity are conducive to early diagnosis and differential diagnosis. 3. The relative independence of the assessments in each cognitive area makes it easier for clinicians to distinguish .4. Low impact of exercise symptoms of PD on detection. 5. Reasonable test time and very low fatigue effect.

Subgroup results, based on different cognitive domains, showed significant improvement on executive function of PD patients after rTMS; especially when high-frequency rTMS stimulation located in the DLPFC for multiple sessions. Similar results have been reported in previous studies. For example, Mogg’s study found that a 10 days of high rTMS posited on DLPFC had some improvement on the executive function of schizophrenia. In addition, Moser et al. [48] performed rTMS stimulation (20.0 Hz and 80% MT) over DLPFC in 19 patients with dysfunction and showed that the Trail making test (TMT) connection test and the Stroop Color and word test (SCWT) scores of the rTMS group had significant improvement. Executive functions include planning, organization, and goal-directed behavioral adjustments. The damage of executive function reflects the damage to the frontal lobes of the brain particularly the DLPFC, and ultimately leads to the degradation of dopaminergic neurons [49]. Low dopaminergic status, such as before dopaminergic therapy, after the removal of levodopa or other dopaminergic drugs, and sudden drug reduction, can cause disorders in executive function which result in reduced flexibility of mental activity [50]. The causes of cognitive impairment in non-demented PD generally include changes in dopaminergic and cholinergic neurotransmitters, neuropathological changes in the limbic system, cortex and other systems, Lewy bodies, neurofibrillary tangles, and cerebrovascular diseases [51]. One previous study [52] found that rTMS stimulated the frontal cortex to regulate the dopamine system, accelerating dopamine release in the basal ganglia which in turn improves the executive function of PD patients. Beyond that, executive function, as a process of higher cognitive function, is usually closely related to the cooperation of multiple brain regions. Perhaps stimulation of the DLPFC not only impacts cortical excitability but also within the stimulated cortex that has been engaged in the cognitive task which leads to excitability changes of the whole circuitry. That is, the associative basal ganglia-thalamo-cortical loop is interconnected with the stimulated area [53, 54].

An important consideration is that the parameters of rTMS are related to effects on cognitive rehabilitation of PD with cognitive deficits. Frequency is one of the most important parameters of rTMS. High frequency can change local neuronal activity and improve the excitability of cerebral cortex. In contrast, low frequency stimulation can inhibit local neuronal activity and reduce the excitability of cerebral cortex. In addition, different frequencies of stimulation may contribute to distinct effects on cortical metabolism and cerebral blood flow. For example, high frequency may lead to increased local metabolism while low frequency may lead to decreased metabolism. As Conca et al. [55] reported, rTMS can change the frontal cerebral blood flow and brain metabolism in patients with depression, thereby improving depressive symptoms. Our results suggest that high frequency stimuli is more effective on cognition, and there have been numerous reports in other psychiatric literature about the efficacy of high frequency for cognitive impairment. For the session of rTMS treatments, a large number of meta-analyses from previous studies have found that treatment sessions have better results within certain limits [56, 57]. In general, rTMS generates local nerves stimulated by micro-currents which affects multi-site functions through the connection and interaction between neural networks. The effect of a single session is limited and hardly long lasting. Multiple sessions results in cumulative and long term benefits. However, excessive stimulations can lead to headaches, nausea, epilepsy, mental disorders and other side effects. Our results showed that the effect of a single session was not significant, but the specific parameters require further experimental support.

Other cognitive domains such as memory, language, and attention have not found significant results. Previous studies of other mental illnesses have shown that rTMS stimulation on the forehead area significantly improved the memory function of patients, but it had not been found in this study. There may be several reasons for this. Firstly clinical manifestations of PD patients were heterogeneous and included memory impairment and non-memory impairment with single lesions and composite lesions. Although some patients showed more memory or cortical injury, in general, single non-memory impairment accounts for the subject. Frontal cortical function or executive function is the most common impaired domain followed by impaired memory function [58, 59]. Another PD-MCI multi-center study showed the similar result that executive function disorders accounted for a large proportion of PD patients with cognitive impairment [7]. Second, the duration of the subjects included in our study was more than 5 years or even longer. Many patients may be in moderate cognitive impairment, and the effect of rTMS is not obvious compared to the MCI. Therefore, although the dorsolateral prefrontal cortex has a significant contribution to memory function, the effect is not significant [60]. For language function, from the past research, the anatomical structure of language function was mainly located in the lower part of the frontal gyrus [61, 62]. Some imaging studies [63] have even found that language dysfunction was related to temporal lobe and language hemispheres. For attention function, the brain regions related to attention are mainly located in parietal and temporal lobes. In the past studies, there have been a lot of similar reports [64, 65] and the damage of the parietal structure can leads to visual neglect [66]. Hilgetag et al. [67] also found that the patient’s visual attention was improved by stimulating the lateral parietal lobe through rTMS. However, most of the stimuli sites included in our study were located in the prefrontal lobe, which may not lead to a corresponding improvement in language and attention function after stimulation. Of course, the completion of any cognitive task is not the result of a single brain region but the product of multiple brain regions. However, the application of accurate rTMS positioning is still a key task in improving different cognitive functions.

There are some shortcomings in this study. First, although 14 articles were included, these results are not all sham-controlled studies which may raise several biases. The current RCTs could minimize the placebo effect, but some neuroimaging techniques have demonstrated that sham-rTMS also can produce considerable placebo effects by inducing striatal dopamine release [68, 69]. Thus, it is difficult to distinguish if the modest improvement is caused by the placebo effect or not. Second, PD patients have different degrees of motor disorders. Some of the PD patients even suffered from moderate or major depression. The intricate intertwined comorbidity is also a problem that cannot be ignored. Third, diversity of assessment tools, and the poor sensitivity and low specificity of some scales may lead to deviations. The cognitive assessment of some articles is only as an additional assessment. In addition, because many articles do not contain important cognitive scales related to the course of PD, we cannot analyze the efficacy of rTMS in these specific areas such as psychomotor speed, visual nerve, etc. Fourth, because the *p*-values were not corrected for multiple comparisons, it may have an impact on the results. Finally, the included studies relatively lacked of the follow-up period, so we could not evaluate the sustainability of its long-term differentiation. Whether the curative effects of rTMS could be sustained for a long time is still unknown. Further studies with large sample sizes of experiments involving long-term follow-up effect after treatment are needed to increase the reliability that rTMS performs on cognitive impairment.

## Conclusion

This study shows rTMS therapy may have a promising effective way of treatment on the cognitive impairment of PD patients. This is particular the case for executive function of PD patients who had benefit with high-frequency rTMS stimulation located in the DLPFC for multiple sessions. In the future, we hope that there will be more experimental design which is rigorous and has large sample experiments to support our results.

## Data Availability

Data generated during this study are included in this published article.

## Abbreviations

rTMS: Repetitive transcranial magnetic stimulation
PD: Parkinson’s disease
SMD: Standardized mean differences
CI: Confidence interval
DLPFC: Dorsolateral prefrontal cortex
AD: Alzheimer’s disease
NMS: Non-motor symptoms
tDCS: Direct current stimulation
RCT: Randomized controlled trials
DRS: Mattis Dementia Rating Scale
MMSE: Mini-Mental State Examination
MoCA: Montreal Cognitive Assessment
SCWT: The Stroop Color and Word Test
TMT: Trail Making Test
